# Supplementary material for: Exercise Counteracts the Deleterious Effects of Cancer Cachexia
Source: Cancers (Basel). 2022 May 19;14(10):2512. doi: 10.3390/cancers14102512 (PMC9139714; doi:10.3390/cancers14102512)
Supplement: Supplementary file 1 [file cancers-14-02512-s001.zip › cancers-1709161-supplementary.pdf]

Supplementary Materials

Exercise Counteracts the Deleterious Effects of Cancer Cachexia

Stavroula Tsitkanou, Kevin A. Murach, Tyrone A. Washington and Nicholas P. Greene

Table S1. Ongoing clinical trials with exercise-based interventions in cancer patients (source: https://clinicaltrials.gov).

Title of Study	Type of Cancer	Location(s) Study Conducts
Lung Cancer Exercise Training Study	Lung Cancer	Memorial Sloan Kettering Cancer Center, New York, New York, United States
Effects of a Physical Exercise and Health Education Program for Women With Breast Cancer Undergoing Chemotherapy	Breast cancer	Hospital Municipal Vila Santa Catarina, São Paulo, Brazil
Part II: Exercise in Hispanic Breast Cancer Survivors	Breast cancer	i) University of Texas MD Anderson Cancer Center, Houston, Texas, United States, ii) University of Puerto Rico, San Juan, Puerto Rico
Precision-exercise-prescription for Lung Cancer Patients Undergoing Surgery: The PEP Study	i) Non Small Cell Lung Cancer, ii) Secondary Lung Cancer	Huntsman Cancer Institute, Salt Lake City, Utah, United States
The Active After Cancer Trial (AACT)	i) Breast Cancer, ii) Colorectal Cancer	i) University of California at San Diego, La Jolla, California, United States, ii) University of California San Francisco, San Francisco, California, United States, iii) Dana-Farber Cancer Institute, Boston, Massachusetts, United States, iv) New Hampshire Oncology Hematology, Concord, New Hampshire, United States, v) Lakes Regional Healthcare Hematology Oncology, Hooksett, New Hampshire, United States, vi) Rosewell Park Cancer Institute, Buffalo, New York, United States, vii) Hematology Oncology Associates of Central New York, East Syracuse, New York, United States, viii) Ohio State University, Columbus, Ohio, United States, ix) Vermont Cancer Center, Burlington, Vermont, United States
CHAMP: A Randomized Controlled Trial of High-intensity Aerobic and Resistance Exercise for Metastatic Prostate Cancer	Prostate Cancer	University of California, San Francisco, San Francisco, California, United States
Randomized Controlled Trial Evaluating the Role of Exercise in Women Undergoing Treatment for Breast Cancer	Non Metastatic Operable Breast Cancer	Tata Memorial Hospital, Mumbai, Maharashtra, India
Pre Radiotherapy Daily Exercise Training in Non-Small Cell Lung Cancer	i) Non-Small Cell Lung Cancer Stage, ii) Chemoradiation	University Hospital of Copenhagen, Copenhagen, Denmark
Doctor-Recommended Home-Based Exercise Program or Relaxation Training in Improving Physical Function and Controlling Symptoms in Patients With Stage IV or Recurrent Colon Cancer That Cannot Be Removed By Surgery	Colorectal Cancer	University of Texas MD Anderson Cancer Center, Houston, Texas, United States
Trial of Exercise to Reduce Cancer Related Fatigue in Breast Cancer	Breast Cancer	Hackensack University Medical Center, Hackensack, New Jersey, United States
Feasibility of Measuring Exercise's Effects on Molecular Mechanisms of Disease Progression in Prostate Cancer	Prostate Cancer	Vancouver Prostate Centre, Vancouver, British Columbia, Canada
Qigong For PPSP In Breast Cancer Pain In Breast Cancer Survivors	Breast cancer	Dana-Farber Cancer Institute, Boston, Massachusetts, United States
UW WELL-FIT Exercise Program for Cancer Patients	N/A	University of Waterloo, Waterloo, Ontario, Canada
Efficacy of Exercise Using Connected Activity Trackers and Therapeutic Education in Localized Breast Cancer	Breast cancer	i) Institut Sainte-Catherine, Avignon, France, ii) CHRU Besançon, Besançon, France, iii) Centre Léon Bérard, Lyon, France
Exercise in Testicular Germ Cell Cancer Survivors	Testicular Germ Cell Tumor	i) National Cancer Institute, Bratislava, Please Select, Slovakia, ii) Biomedical Research Center Slovak Academy of Sciences, Bratislava, Please Select, Slovakia

A Single Arm Pilot Study to Refine a Novel Approach to Exercise Promotion Based on Affect-regulation	Breast Cancer	Dartmouth-Hitchcock Health, Lebanon, New Hampshire, United States
Physical Activity, Proliferation and Immune Markers in Benign Breast Tissue	Breast Cancer	Dana Farber Cancer Institute, Boston, Massachusetts, United States
Supervised Aerobic Training During or After Chemotherapy for Operable Breast Cancer	Breast Cancer	Memorial Sloan Kettering Cancer Center, New York, New York, United States
CanMove: A Physical Activity Program for Children With Cancer	Pediatric Cancer	Royal Children's Hospital, Parkville, Victoria, Australia
Physical Activity for Advanced Stage Cancer Patients	N/A	H. Lee Moffitt Cancer Center and Research Institute, Tampa, Florida, United States
Diet, Exercise and Vitamin D in Breast Cancer Recurrence	Breast Cancer	i) Centro Riferimento Oncologico, Aviano, Italy, ii) Azienda Ospedaliera Cannizzaro, Catania, Italy, iii) Clinica Mediterranea, Napoli, Italy, iv) Istituto Nazionale Tumori Fondazione G. Pascale, Napoli, Italy, v) Ospedale dei Colli Monaldi, Napoli, Italy, vi) Ospedale Evangelico Betania, Napoli, Italy, vii) Ospedale San Vincenzo, Taormina, Italy
Physical Activity in Patients With Metastatic Colorectal Cancer Who Receive Palliative First-line Chemotherapy	Metastatic Colorectal Cancer	i) Universitätsklinikum der PMU Salzburg, Salzburg, Austria, ii) Klinikum Wels-Grieskirchen GmbH, Wels, Austria, iii) Tumor Zentrum Aarau, Aarau, Switzerland, iv) Kantonsspital Aarau, Aarau, Switzerland, v) Kantonsspital Baden, Baden, Switzerland, vi) St. Claraspital, Basel, Switzerland, vii) Clinical Cancer Research Center at University Hospital Basel, Basel, Switzerland, viii) Istituto Oncologico della Svizzera Italiana IOSI, Bellinzona, Switzerland, ix) Spitalzentrum Biel, Biel, Switzerland, x) Spitalzentrum Oberwallis, Brig, Switzerland and 14 more
Exercise Intervention in Preventing Breast Cancer Recurrence in Postmenopausal Breast Cancer Survivors	Breast Cancer	City of Hope Medical Center, Duarte, California, United States
The Preoperative Health & Body Study	Breast Cancer	Dana-Farber Cancer Institute, Boston, Massachusetts, United States
Intensive Diet and Exercise or Standard of Care in Improving Physical Function and Quality of Life in Patients With Prostate Cancer Undergoing Androgen Deprivation Therapy	Prostate Cancer	Arthur G. James Cancer Hospital and Solove Research Institute at Ohio State University Medical Center, Columbus, Ohio, United States
The SEHNeCa Supervised Exercise Project	Neack and Head Cancer	Biocruces Bizkaia research health institute, Barakaldo, Bizkaia, Spain
Exercise Therapy in Radiation Therapy	Metastatic Cancer	Penn State Cancer Institute, Hershey, Pennsylvania, United States
FOcus on Reducing Dose-limiting Toxicities in Colon Cancer With Resistance Exercise Study	Colon Cancer	i) Kaiser Permanente Division of Research, Oakland, California, United States, ii) Dana Farber Cancer Institute, Boston, Massachusetts, United States, iii) Penn State Cancer Institute, Hershey, Pennsylvania, United States
Trial Comparing the Effects of Linear Versus Nonlinear Aerobic Training in Women With Operable Breast Cancer	Breast Cancer	Memorial Sloan Kettering Cancer Center, New York, New York, United States
Energy Balance and Breast Cancer Aspects-II	Breast Cancer	i) Oslo University Hospital, Oslo, Norway, ii) St. Olavs Hospital, Trondheim, Norway
Development of Patient Tailored Guideline of Physical Activity for Lung Cancer	Lung Cancer	Samsung Medical Center, Seoul, Korea
Methylphenidate and Physical Activity to Reduce Cancer Related Fatigue Due to Anti PD1 Immunotherapy	Malignant Neoplasm	M D Anderson Cancer Center, Houston, Texas, United States
Narrative Visualization for Breast Cancer Survivors' Physical Activity	Breast Cancer	The University of Texas Medical Branch, Galveston, Texas, United States
An Early Stress-Reduction Intervention in Patients With Newly Diagnosed Breast Cancer	Malignant Neoplasm of Breast	University of Texas MD Anderson Cancer Center, Houston, Texas, United States
Adapted Physical Activity (APA) in a Breast Cancer Population.	Breast Cancer	University of Perugia, Perugia, Italy
Physical Activity and Dexamethasone in Reducing CancerRelated Fatigue in Patients With Advanced Cance	Malignant Neoplasm of Breast	M D Anderson Cancer Center, Houston, Texas, United States
The Effects of Moderate Exercise on Distress, Quality of Life, and Biomarkers of Angiogenesis and Chronic Stress in Ovarian Cancer Survivors	Ovarian Cancer	Fred Hutch/University of Washington Cancer Consortium, Seattle, Washington, United States
High-Flow Oxygen in Reducing Shortness of Breath Caused by Exercise in Patients With Cancer	Malignant Neoplasm	M D Anderson Cancer Center, Houston, Texas, United States

Physical Training and Cancer-a Multicenter Clinical Trial	i) Breast Cancer, ii) Colorectal Cancer, iii) Prostate Cancer	i) Uppsala University, ii) The Swedish Research Council, iii) Swedish Cancer Society, iv) Nordic Cancer Union, v) Linkoeping University, vi) Lund University, vii) University of Agder, viii) Copenhagen University Hospital-Denmark, ix) Norwegian School of Sport Sciences, x) Amsterdam UMC-location VUmc and 2 more
Web-Based Physical Activity Intervention in Improving Long Term Health in Children and Adolescents With Cancer	i) Carcinoma In Situ, ii) Hematopoietic and Lymphoid System Neoplasm, iii) Malignant Solid Neoplasm	i) Children's Hospital of Alabama, Birmingham, Alabama, United States, ii) Phoenix Childrens Hospital, Phoenix, Arizona, United States, iii) Banner University Medical Center - Tucson, Tucson, Arizona, United States, iv) Arkansas Children's Hospital, Little Rock, Arkansas, United States, v) Kaiser Permanente Downey Medical Center, Downey, California, United States, vi) City of Hope Comprehensive Cancer Center, Duarte, California, United States, vii) Miller Children's and Women's Hospital Long Beach, Long Beach, California, United States, viii) Valley Children's Hospital, Madera, California, United States, ix) UCSF Benioff Children's Hospital Oakland, Oakland, California, United States, x) Kaiser Permanente-Oakland, Oakland, California, United States and 102 more
Game-Based Physical Activity in Childhood Cancer Survivors	Childhood Cancer	Memorial Sloan Kettering Cancer Center, New York, New York, United States
Physical Activity in Relation to Surgical Procedures	Colorectal Cancer	i) Sahlgrenska University Hospital/Östra, Göteborg, Sweden, ii) Dept. of Surgery, Skaraborgs Sjukhus, Skövde, Sweden
Feasibility of Lifestyle Intervention in BRCA1/2 Mutation Carriers	Hereditary Breast and Ovarian Cancer	i) University of Cologne, Cologne, Germany, ii) University of Schleswig-Holstein Campus Kiel, Kiel, Germany, iii) Technische Universitaet Muenchen, Munich, Germany
Health and Recovery Program in Increasing Physical Activity Level in Stage IA-IIIA Endometrial Cancer Survivors	Endometrial Carcinoma	i) Stanford University, School of Medicine, Palo Alto, California, United States, ii) Stanford Cancer Center South Bay, San Jose, California, United States
Multimodal Therapy for the Treatment of Fatigue in Patients With Prostate Cancer Receiving Radiotherapy With Androgen Deprivation Therapy	Prostate Cancer	University of Texas MD Anderson Cancer Center, Houston, Texas, United States
Prehabilitation for Women Undergoing Pre-operative Chemotherapy for Breast Cancer	Breast Cancer	Sunnybrook Health Sciences Centre, Toronto, Ontario, Canada
Lifestyle, Exercise, and Nutrition Study Early After Diagnosis	Breast Neoplasms	i) Yale University, New Haven, Connecticut, United States, ii) Dana Farber Cancer Center, Boston, Massachusetts, United States
Pink Warrior 2: Teleconference-based Gaming Support	Breast Cancer	i) The University of Texas Medical Branch, Galveston, Texas, United States, ii) UT MD Anderson Cancer Center, Houston, Texas, United States
The Patterns of Activity and Cognition During Treatment (PACT) Study	Breast Cancer	University of Nebraska Medical Center, Omaha, Nebraska, United States
Multimodal Intervention for Cachexia in Advanced Cancer Patients Undergoing Chemotherapy	Neoplasms	i) Cedars-Sinai Medical Center, Los Angeles, California, United States, ii) CA4 Brampton Civic Hospital, Brampton, Canada, iii) Cross Cancer Insitute, Edmonton, Canada, iv) Jewish General Hospital, Montréal, Canada, v) Ottawa Regional Cancer Centre, Ottawa, Canada, vi) Universitätsklinikum Bonn, Bonn, Germany, vii) Oslo University Hospital, Oslo, Norway, viii) St Olavs Hospital, Trondheim, Norway, ix) Tumor Zentrum, Aarau, Switzerland, x) Cantonal Hospital, St. Gallen, Switzerland and 7 more
Feasibility of the NEXT Steps Weight Loss Intervention +/- Resistance Training for Endometrial Cancer Survivors: Effect on Lean Mass & Biomarkers	Malignant Neoplasms of Female Genital Organs	University of Texas MD Anderson Cancer Center, Houston, Texas, United States
Exercise Intervention for Bone Tumor Patients	Bone Sarcoma	University Hospital Essen, Essen, NRW, Germany
Anamorelin Hydrochloride, Physical Activity, and Nutritional Counseling in Decreasing Cancer-Related Fatigue in Patients With Incurable Metastatic or Recurrent Solid Tumors	Metastatic or Recurrent Malignant Solid Neoplasm	M D Anderson Cancer Center, Houston, Texas, United States
Avanzando Juntas: Adapting an Evidence Based Weight Loss Program for Hispanic Breast Cancer Survivors	i) Breast Cancer, ii) Gynecologic Cancer	Medical College of Wisconsin, Milwaukee, Wisconsin, United States
Exercise Effect on Chemotherapy-Induced Neuropathic Pain	i) Breast cancer, ii) Colorectal cancer, iii) Lung cancer, iv) Ovarian cancer	Baltimore VA Medical Center, Baltimore, Maryland, United States
Aerobic Training in Metastatic Breast Cancer	Metastatic Breast Cance	i) Memorial Sloan Kettering Cancer Center, New York, New York, United States, ii) Duke University Medical Center, Durham, North Carolina, United States
Lifestyle, Exercise and Nutrition Study 2 (LEAN 2)	Breast Cancer	Yale University, New Haven, Connecticut, United States
Develop and Test the 'Multi-Faced Continuous Exercise Program' in Operable Head and Neck Cancer Patients	Head and Neck Squamous Cell Carcinoma	National Taiwan University Hospital

Role of Individualized Intervention(s) in Hormone-Receptor Positive Early-stage Breast Cancer	Breast Cancer	i) Allan Blair Cancer Center, Regina, Saskatchewan, Canada, ii) Saskatoon Cancer Center, Saskatoon, Saskatchewan, Canada
Gentle Yoga and Dietary Counseling in Improving Physical Function and Quality of Life in Stage I-II Endometrial Cancer Survivors	Endometrial Carcinoma	Arthur G. James Cancer Hospital and Solove Research Institute at Ohio State University Medical Center, Columbus, Ohio, United States
The Prehabilitation Study: Exercise Before Surgery to Improve Patient Function in People	i) Intraabdominal cancer, ii) Thoracic cancer	The Ottawa Hospital, General Campus, Ottawa, Ontario, Canada
Cycling in Preventing Colorectal Cancer in Participants With Lynch Syndrome	Lynch Syndrome	M D Anderson Cancer Center, Houston, Texas, United States
Heal-Me Personalized Online Nutrition and Exercise Routines	N/A	University of Alberta, Edmonton, Alberta, Canada
Activity Program During Aromatase Inhibitor Therapy	Early Breast Cancer	i) Kantonsspital Aarau, Aarau, Switzerland, ii) Kantonsspital Baden, Baden, Switzerland, iii) CABA - Zentrum für Onkologie, Psychologie und Bewegung, Basel, Switzerland, iv) Brustzentrum Basel - Praxis für ambulante Tumortherapie, Basel, Switzerland, v)Universitätsspital Basel, Basel, Switzerland, vi) Inselspital Bern, Bern, Switzerland, vii) Hirslanden Brustzentrum Bern Biel, Bern, Switzerland, viii) Kantonsspital Graubünden, Chur, Switzerland, ix) Tumorzentrum ZeTuP Chur, Chur, Switzerland, x) Clinique des Grangettes, Centre du sein, Chêne-Bougeries, Switzerland and 22 more
Lifestyle Change for Better Health	i) Bladder Cancer, ii) Colorectal Adenoma	UPMC Shadyside, Pittsburgh, Pennsylvania, United States
Prostate Cancer - Patient Empowerment Program	Prostate Cancer	QEII Health Sciences Centre, Halifax, Nova Scotia, Canada
Tai Chi for Relieving Aromatase Inhibitor-Induced Arthralgia in Patients With Stage I-III Breast Cancer, the TaiChi4Joint Trial	Breast Cancer	Thomas Jefferson University Hospital, Philadelphia, Pennsylvania, United States
Sensorimotor Rehabilitation Program in Improving Quality of Life in Patients With Early Stage Breast Cancer	Breast Cancer	Ohio State University Comprehensive Cancer Center, Columbus, Ohio, United States
Exercise Prescription for the Improvement of Quality of Life in Elderly Patients With Multiple Myeloma	Plasma Cell Myeloma	Sidney Kimmel Cancer Center at Thomas Jefferson University, Philadelphia, Pennsylvania, United States
Weight Management and Health Behavior Intervention in Lowering Cancer Risk for BRCA Positive and Lynch Syndrome Families	i) Hereditary Breast Carcinoma, ii) Hereditary Ovarian Carcinoma, iii) Lynch Syndrome	M D Anderson Cancer Center, Houston, Texas, United States
The Role of Lifestyle Factors in Breast Cancer-Related Outcomes	Breast Cancer	M D Anderson Cancer Center, Houston, Texas, United States
Exercise and QUality Diet After Leukemia: The EQUAL Study	Adult Survivors of Childhood Leukemia	i) Memorial Sloan Kettering Cancer Center, New York, New York, United States, ii) St. Jude Children's Research Hospital Memphis, Tennessee, United States
Tibetan Yoga in Improving Fatigue and Sleep in Participants With Stage I-III Breast Cancer	Breast Cancer	M D Anderson Cancer Center, Houston, Texas, United States
MoveStrong at Home	N/A	University of Waterloo, Waterloo, Canada
